# Outcomes of Small Size Ahmed Glaucoma Valve Implantation in Asian Chronic Angle-Closure Glaucoma

**DOI:** 10.3390/jcm10040813

**Published:** 2021-02-17

**Authors:** Ke-Hao Huang, Ching-Long Chen, Da-Wen Lu, Jiann-Torng Chen, Yi-Hao Chen

**Affiliations:** 1 Department of Ophthalmology, Tri-Service General Hospital, National Defense Medical Center, Taipei 114, Taiwan; a912572000@gmail.com (K.-H.H.); doc30881@mail.ndmctsgh.edu.tw (C.-L.C.); ludawen@yahoo.com (D.-W.L.); jt66chen@gmail.com (J.-T.C.); 2 Department of Ophthalmology, Song-Shan Branch of Tri-Service General Hospital, National Defense Medical Center, Taipei 105, Taiwan

**Keywords:** Ahmed glaucoma valve, angle closure glaucoma, surgical modification, surgical outcome

## Abstract

For chronic angle-closure glaucoma (ACG), Ahmed glaucoma valve (AGV) is a useful drainage device for intraocular pressure (IOP) control but there are few reports discussing the outcomes of small size AGV in adult patients. This retrospective study involved 43 Asian adult patients (43 eyes) with chronic ACG. All patients had undergone small size AGV insertion and were divided into anterior chamber (AC) group and posterior chamber (PC) group. In the AC group, tube was inserted through sclerectomy gap into the anterior chamber. In the PC group, tube was inserted into posterior chamber through a needling tract. Outcome measures were intraocular pressure (IOP), visual acuity, number of antiglaucoma medications, survival curve and incidence of complications. In total, 43 eyes of 43 patients, 24 in the AC group and 19 in the PC group, were reviewed. The mean follow-up period was 28.5 months (95% confidence interval: 25.5–31.4). Mean IOP had significantly decreased following AGV insertion. The Kaplan–Meier survival analysis demonstrated a probability of success at 24 months of 67.4% for qualified success and 39.5% for complete success. There were no significant differences between the AC and PC groups in terms of the mean IOP, cumulative probability of success, visual acuity change or antiglaucoma medication change, except IOP at 1-day and 1-month mean IOP. The most common complications noted was hyphema in the PC group. For adult chronic ACG patients, small size AGV insertion could be effective at lowering IOP. Besides, tube insertion into AC with sclerectomy may prevent the hypertensive phase in the early postoperative period.

## 1. Introduction

Angle closure glaucoma (ACG) is caused by impaired outflow facility secondary to appositional or synechial closure of the anterior chamber drainage angle, which leads to elevated intraocular pressure (IOP), optic nerve damage and visual field loss. Based on etiology, ACG can be divided into primary or secondary ACG. In Asian populations, primary ACG is estimated to pose a greater risk of blindness than primary open-angle glaucoma [1]. The gold standard to treat ACG is reducing IOP by medication, peripheral iridotomy and controlling the underlying causes. However, some eyes are resistant to these treatments and thus surgical intervention may be necessary. Several surgical procedures, such as anterior chamber paracentesis, surgical iridectomy, simple lens extraction, trabeculectomy or a combination of lens extraction and trabeculectomy are utilized for treating ACG and different procedures are chosen according to patient’s condition.

Implantation of Ahmed glaucoma valve (AGV) is effective to control IOP in glaucoma patients and there are two different size models for adult and children, respectively. The difference is the surface area, which is 184 mm^2^ for adult and 96 mm^2^ for children. AGV is indicated commonly for the patients who had failed previous trabeculectomy. In such cases, the availability of viable conjunctiva would be reduced and the risk of complications like conjunctival buttonhole, wound leak and tube or plate exposure may increase. In glaucoma patients with failed previous trabeculectomy, small size AGV may be beneficial to make AGV implantation possible because it can reduce the need of viable conjunctiva during surgery. Besides, data on AGV insertion in ACG are limited because of the relative high risk of corneal complications such as shallow anterior chamber and tube-corneal touch [2,3]. Previously, tube insertion via the ciliary sulcus or pars plana was reported to avoid these complications [4,5] but this technique cannot be performed easily and was not applicable in all cases of ACG. Therefore, evaluation of the outcomes of small size AGV in adult chronic ACG patients may provide useful information.

In the present study, we reported the results of small size AGV insertion in adult chronic ACG patients. And we also evaluated the benefit and safety of modified technique (sclerectomy) for AGV tube insertion to anterior chamber.

## 2. Materials and Methods

### 2.1. Patients

This retrospective review was carried out at a tertiary referral medical center in northern Taiwan between 2009 and 2014. The study was approved by the institutional review board of Tri-Service General Hospital, Taipei, Taiwan (TSGHIRB No. 1-103-05-144), which waived the requirement for informed consent from participants and allowed access to the follow-up clinical records. It was conducted in accordance with the requirements of the Declaration of Helsinki. Patients with chronic ACG with an uncontrolled IOP who had undergone a small size AGV insertion were included. Chronic ACG was defined by the following criteria: patients had surgical history of previously failed trabeculectomy and the angle presentation of the eye was lower than grade 2 before surgery, according to the Shaffer gonioscopic grading system and with glaucomatous changes including abnormal optic disc appearance, loss of the nerve fiber layer and visual field defects. Uncontrolled IOP was defined as an IOP > 21 mmHg, even after maximal usage of antiglaucoma medications. Exclusion criteria included primary angle-closure suspect, primary angle closure with no optic nerve damage, any ocular surgeries within the previous 3 months, a follow-up period less than 2 years, a preoperative visual acuity (VA) lower than light perception or were younger than 20 years of age. Besides, the decision of small size AGV implantation is made by surgeon during surgery, based on the conjunctival mobility, scarring due to previous surgery like trabeculectomy and limited surgical area which may be due to conjunctival scarring or small fissure height that even the maximum extension is made by lid speculum and traction suture. This study enrolled 46 chronic ACG patients. 3 of them were lost to follow up and were excluded from the analysis. In the remaining 43 patients, 24 patients were in the anterior chamber (AC) group and the other 19 patients were in the posterior chamber (PC) group, which was based on the presence of anterior synechiae at tube insertion site or not.

Patients’ demographic characteristics, namely best-refracted VA, IOP, number of antiglaucoma medications, type of chronic ACG and history of ocular surgery, systemic diseases or complications were recorded and assessed by a chart review. VA was measured via a standard Snellen chart and IOP via the Goldmann applanation tonometer. Defining the time of operation as the baseline, each patient was followed up 1 day, 2 weeks, 1 month, 3 months, 6 months, 1 year, 2 years and 3 years after surgery.

### 2.2. Outcome Measures

Mean IOP, vision changes, complications and the number of antiglaucoma medications taken after the Ahmed glaucoma valve insertion were evaluated as the outcome measures. Qualified success was defined by an IOP < 21 mmHg with or without antiglaucoma medications; complete success was defined by an IOP < 21 mmHg without additional therapy; failure was defined by (1) an IOP > 21 mmHg despite the use of antiglaucoma medications at 2 consecutive visits, (2) light perception negative vision, (3) requirement of additional glaucoma surgery or (4) devastating operative or postoperative complications such as endophthalmitis or phthisis.

### 2.3. Surgical Technique

**Figure 1:**
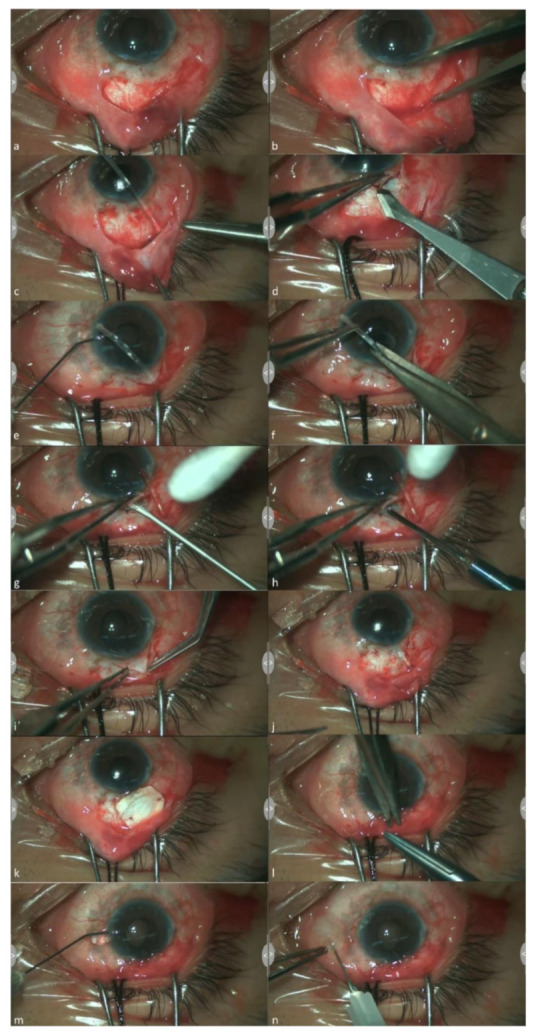
Surgical technique description. (**a**) fornix-based conjunctival flap was created in the superotemporal quadrant between two adjacent recti muscles under general anesthesia. (**b**) Ahmed plate would be placed 7 mm posterior to the corneoscleral limbus. (**c**) Ahmed plate is fixed firmly to the sclera with 8-0 prolene sutures. (**d**). 6 mm limbus-based triangular partial thickness scleral flap was created. (**e**) anterior chamber paracentesis was made by microvitreoretinal (MVR) blade and viscoelastic was injected through the paracentesis. (**f**) the tube was trimmed. (**g**) anterior chamber incision at limbus was made by MVR blade. (**h**) sclerectomy gap was made by Kelly Descemet’s membrane punch. (**i**) the residual length of tube was modified as desired (about 1–2 mm in anterior chamber). (**j**) the scleral flap was closed with 10-0 nylon sutures and the tube was secured with 8–0 prolene suture. (**k**) A human donor scleral graft was placed on the tube with the anterior edge adjacent to the limbus and sutured to the sclera with an 8-0 Vicryl suture. (**l**) The conjunctiva was then re-approximated with 8-0 Vicryl interrupted sutures. (**m**) viscoelastic anterior chamber injection was repeated to avoid early postoperative hypotony. (**n**) subconjunctival injection of dexamethasone and gentamicin.

Surgical and postoperative management procedures were similar across all patients and the procedures were shown in Figure 1.

In each case, a fornix-based conjunctival flap was created in the superotemporal or superonasal quadrant between two adjacent recti muscles under general anesthesia. The AGV (model S3 or FP-8, New World Medical, Rancho Cucamonga, CA, USA) was irrigated with balanced saline solution (BSS, Alcon, Fort Worth, TX, USA) to prime the valve mechanism. The plate was soaked with 0.4 mg/mL mitomycin-C and then was wiped dry 10 s later. And it was placed at least 7 mm posterior to the corneoscleral limbus and fixed firmly to the sclera with 8-0 prolene sutures (Ethicon Inc., Somerville, NJ, USA). A 6 mm limbus-based triangular partial thickness scleral flap was created at the site of tube incision. Anterior chamber paracentesis was made by microvitreoretinal (MVR) blade and about 0.1 mL viscoelastic (Healon GV, Advanced Medical Optics, Santa Ana, CA, USA) injection was performed through the paracentesis. The tube was trimmed and the residual length was left as desired.

In AC group, anterior chamber incision was made by MVR blade under the scleral flap through the limbus and then a sclerectomy gap was made by Kelly Descemet’s membrane punch. The tube in the anterior chamber was left a length of 1–2 mm lying on the iris and away from the corneal endothelium.

**Figure 2:**
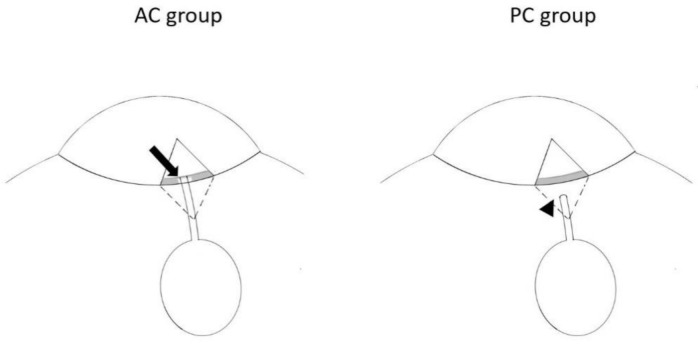
The difference between anterior chamber (AC) group and posterior chamber (PC) group. In the AC group, the tube was inserted into anterior chamber through the sclerectomy gap (arrow) under partial thickness scleral flap. In the PC group, the tube was inserted to ciliary sulcus (arrowhead; 1.5 mm posterior to the limbus) through 23 G needling tract.

The decision of tube insertion to AC or PC is based on the condition of the tube insertion site. If there were possible complications for AC insertion like tube–endothelial contact, patients with anterior synechiae at tube insertion site would be arranged to PC group. In PC group, the tube was inserted to ciliary sulcus (1.5 mm posterior to the limbus) through a tract which is made by 23 G needle without sclerectomy. It is not possible to perform sclerectomy in PC group due to the anatomical consideration, which ciliary body could be damaged after sclerectomy. The difference between AC and PC group was shown in Figure 2.

After the tube was inserted, the superficial scleral flap was closed tightly with 10-0 nylon sutures (Ethicon Inc., Somerville, NJ, USA). And the tube was secured with 8-0 prolene suture. A human donor scleral graft was placed on the tube with the anterior edge adjacent to the limbus and sutured to the sclera with an 8-0 Vicryl suture (Ethicon Inc., Somerville, NJ, USA). The conjunctiva was then re-approximated with 8-0 Vicryl interrupted sutures. And then 0.2 mL viscoelastic anterior chamber injection was repeated to avoid early postoperative hypotony. In the end of the surgery, a subconjunctival injection of dexamethasone and gentamicin was administered.

All surgeries were performed by the authors, DW Lu and YH Chen. After the operation, topical antibiotic (tobramycin or norfloxacin) and steroid (prednisolone acetate) eye drops were applied routinely for 4–8 weeks and tapered gradually. Viscoelastic anterior chamber reformation was performed under a slit-lamp if early flat chamber was noted after surgery. Antiglaucoma medications were adjusted based on IOP and the clinical status of the operated eye.

### 2.4. Statistical Methods

All data are presented as mean (95% confidence interval (CI)). The distributions of variables in the 2 groups were compared using the unpaired Student’s t test for continuous variables and the chi-square or Fisher exact test for categorical data. IOP values were compared by the Wilcoxon signed-rank test or Mann-Whitney U test. In addition, post-hoc power was calculated if significant IOP difference was noted assuming a two-sided alpha = 0.05 for the fixed sample size (*n* = 43) of the present study. A Kaplan-Meier life table analysis was conducted to access the survival rates of the surgical method. *p* < 0.05 was considered statistically significant. All statistical analyses were performed using GraphPad Prism 5.0 (GraphPad Software, Inc., San Diego, CA, USA).

## 3. Results

In total, 43 patients with chronic ACG were included in this study: 24 patients in the AC group and 19 in the PC group. The demographic characteristics of the total patient population and of the AC and PC groups are shown in Table 1. Twenty-four eyes had undergone prior trabeculectomies and twenty eyes had undergone prior cataract surgeries in the AC group; all eyes had undergone prior trabeculectomies and cataract surgeries in the PC group. All included patients did not have surgical history of vitrectomy. The mean follow-up time of all patients were 29.5 months (95% CI: 24.8–34.2) for the AC group and 27.2 months (95% CI: 23.5–30.8) for the PC group.

**Figure 3:**
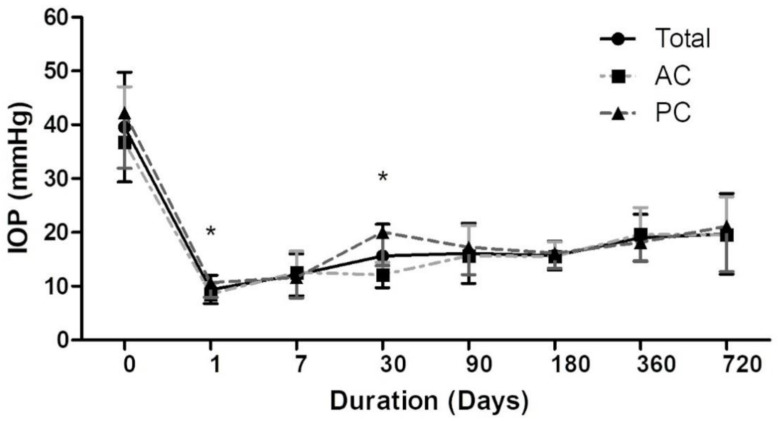
Preoperative and postoperative intraocular pressure (IOP) after Ahmed glaucoma valve implantation surgery plotted over time. Addressed IOP data includes successful and failed patients. AC: anterior chamber; PC: posterior chamber. *: *p* < 0.05.

Table 2 represents mean IOP data for all patients, not only successful but also failed patients’ IOP data were collected at all time intervals since preoperative to postoperative follow-up period. Compared with the baseline value, IOP differed significantly over time (*p* < 0.05) in the total patient population and in both the AC and PC groups. There were no significant differences between the AC and PC groups at any of the time points except at 1-day (*p* = 0.0160) and 1-month (*p* < 0.0001) post-surgery. The mean IOP at 1-day and 1-month post-surgery was 8.5 mmHg (95% CI: 7.6–9.4) and 12.1 mmHg (95% CI: 11.2–13.1) in the AC group and 10.6 mmHg (95% CI: 9.3–11.9) and 20.0 mmHg (95% CI: 17.0–23.0) in the PC group, respectively. As shown in Figure 3, IOP was noted to be significantly higher in the PC group than in the AC group at 1-day and 1-month post-surgery. Post-hoc statistical power for detecting the IOP difference was 76.8% for 1-day IOP and 99.9% for 1-month IOP.

**Figure 4:**
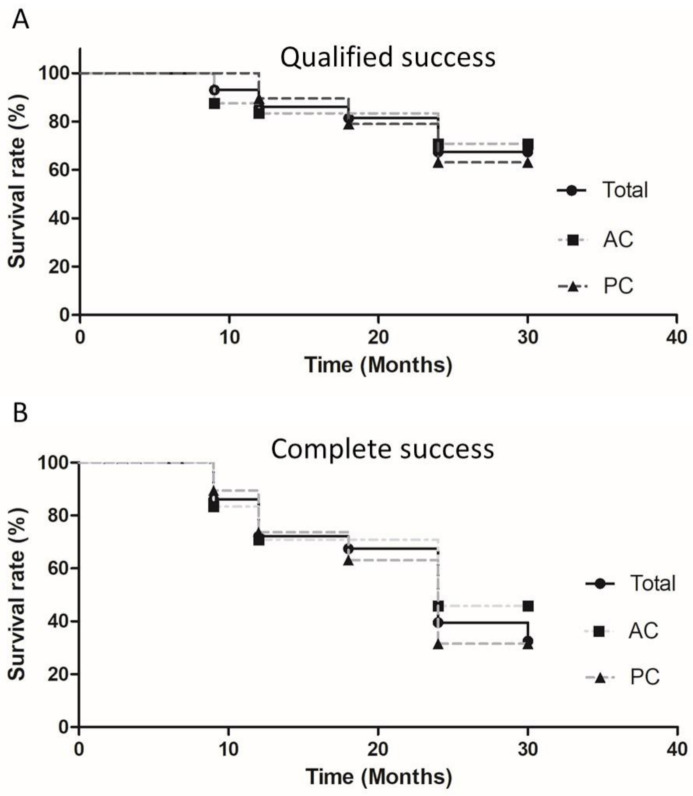
Kaplan-Meier curve showing the probability of qualified success (**A**) (*p* = 0.67, log rank test for comparing AC and PC surviving curves) and complete success (**B**) (*p* = 0.43, log rank test for comparing AC and PC surviving curves) after Ahmed glaucoma valve implantation surgery. AC: anterior chamber; PC: posterior chamber.

The Kaplan-Meier survival curves for qualified success of the total patient population and the AC and PC groups are shown in Figure 4A. The probability of qualified success was 67.4% at 24 months for all patients (AC group versus PC group: 70.8% versus 63.2%). No significant differences (*p* = 0.67, log rank test) were noted between the AC and PC groups. Of the 7 failed eyes in the AC group, 5 underwent second AGV insertions, 1 underwent evisceration of the eyeball and 1 underwent a cyclodestructive procedure. Of the 7 failed eyes in the PC group, 5 underwent second AGV implantations and 2 underwent cyclodestructive procedures. When complete success was used as the criterion, the Kaplan-Meier survival curves for the total patient population and the AC and PC groups are shown in Figure 4B. The probability of complete success was 39.5% at 24 months for all patients (AC group versus PC group: 45.8% versus 31.6%). There were no significant differences (*p* = 0.43, log rank test) between the AC and PC groups.

**Figure 5:**
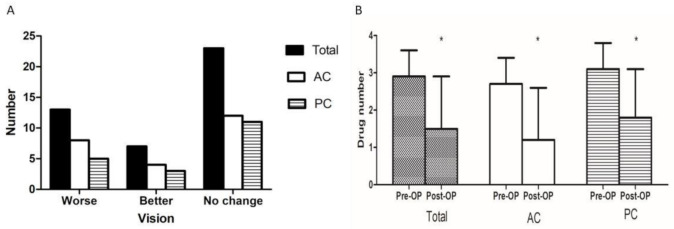
Changes in vision (**A**) and number of antiglaucoma medications (**B**) between the baseline and the final visit. AC: anterior chamber; PC: posterior chamber; OP: operation. *: *p* < 0.05.

A comparison of the final changes in the vision of the total patient population and both the AC and PC groups is shown in Figure 5A. There were no significant differences between the AC and PC groups with respect to “worse,” “better” or “no change” classifications. In the AC group, 12 patients (50%) showed no change in vision, 4 (16.7%) showed an improvement and 8 (33.3%) showed a decline. Similarly, 11 patients in the PC group (57.9%) showed no change in vision, 3 (15.8%) showed an improvement and 5 (26.3%) showed a decline (Table 3).

In terms of changes in antiglaucoma medication, the number of antiglaucoma drugs used preoperatively and postoperatively are shown in Figure 5B. In the total patient population and both the AC and PC groups, significant differences were noted between the number of preoperative and postoperative medications (Table 4). The average number of antiglaucoma medications used prior to AGV implantation was 2.7 (95% CI: 2.4–3.0) in the AC group and 3.1 (95% CI: 2.8–3.5) in the PC group. This difference between the two groups was not statistically significant. After AGV implantation, the mean number of medications used was 1.2 (95% CI: 0.6– 1.8) in the AC group and 1.8 (95% CI: 1.2–2.5) in the PC group. This difference between the two groups was also not statistically significant.

No intraoperative complications were noted in this study and the postoperative complications are listed in Table 5. The early postoperative complications occurring in the first month were hyphema, flat chamber/hypotony and tube or plate exposure. The frequency of hyphema was significantly higher in the PC group than in the AC group (*p* < 0.05) and this complication was resolved spontaneously. Although there was no statistically significant difference in the complication of flat chamber/hypotony, a higher incidence of viscoelastic anterior chamber reformation was 0.5 more in the AC group (0.9 ± 0.7 vs. 0.4 ± 0.8; *p* < 0.05). The late postoperative complications, which occurred more than 3 months post-surgery, were tube retraction, encapsulated bleb, endophthalmitis, bullous keratopathy and strabismus. There were no significant differences between the AC and PC groups with respect to these late complications. More than one complication was noted in some eyes.

## 4. Discussion

ACG accounts for a large proportion of glaucoma cases in Asia [6] and needs to be adequately managed. The strategy for treating ACG is releasing the pupillary block using laser peripheral iridotomy and maintain IOP with or without medication. Glaucoma surgery is performed to lower IOP when further IOP reduction cannot be achieved by medical or laser treatment. The implantation of a glaucoma drainage device is one surgical option. One example of such a device is an AGV, which consists of a flow resistance valve. AGV insertion has been reported in uveitic and pediatric glaucoma with varying results [7,8]. In past reports, mean IOP was maintained below the teens and the cumulative probability of success was 68–75% at 2 years after AGV implantation [3,9]. Our results were similar to those of previous studies: mean IOP decreased to 9.4 mmHg at day 1 and 20.2 mmHg at 2 years and the cumulative probability of qualified success was 67.4% at 2 years after AGV implantation. In most eyes (69.8%), Vision was maintained or improved after AGV implantation. The mean number of antiglaucoma medications also decreased from 2.9 to 1.5 after AGV implantation. Although the development of narrow angle in uveitic, neovascular or post-penetrating keratoplasty glaucoma is associated with poor prognosis with respect to IOP control, our study demonstrated that the results of AGV implantation in chronic ACG may not be inferior to the results in other glaucoma types.

Therapeutically, the success of IOP control after tube shunt surgery is related to the size of the plate, plate material, profile, surface texture and the severity of surgical damage [10]. IOP reduction is greater when a larger plate is used. However, in our study, the cumulative probability of success of AGV implantation was not inferior to other studies although we used the small size AGV. East Asian individuals are considered to have small eyes, puffy eyelids and shallow orbits [11,12]. Besides, in our study, all the included patients have some extent of conjunctival scarring and limited surgical view. In these individuals, therefore, implantation of adult-sized AGVs may be difficult to manipulate and may cause more complication like conjunctival buttonhole, wound leak and tube or plate exposure and strabismus. Small size AGV implantation may decrease tissue damage and then lower the severity of surgical trauma and scar formation in these patients. In our research, small size AGV implantation could also be effective at lowering IOP for Asian adult chronic ACG patients, exhibiting an acceptable cumulative probability of success and less ocular tissue damage.

**Figure 6:**
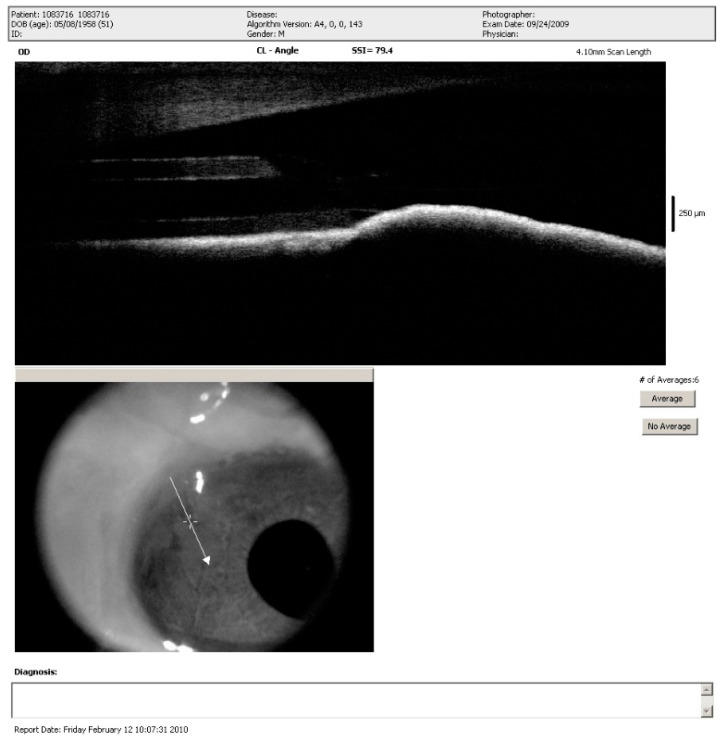
Image of sclerectomy-combined Ahmed glaucoma valve implantation into the anterior chamber by anterior optical coherence tomography, showing the tube lying on the iris.

The conventional procedure of AGV implantation involves puncture of the anterior chamber by a needle for tube insertion. However, this is difficult in eyes with ACG because tube in shallow anterior chamber may damage adjacent tissues such as the cornea or iris. Therefore, AGV implantation is relatively contraindicated in ACG. To reduce this risk, tube insertion through the posterior chamber or pars plana has been reported. However, tube insertion through the posterior chamber cannot be performed in phakic eyes and insertion through the pars plana is a difficult technique. In this study, we used sclerectomy instead of needle puncture in the AC group and the sclerectomy gap could provide a larger space for tube insertion without injuring adjacent tissue. Moreover, we left a relative short length of the tube (1–2 mm) in the AC group. We believe that such modification could make the tube lie on the iris and reduce the chance of tube-endothelium contact, as shown in Figure 6. In our series, the incidence of bullous keratopathy did not differ between the AC and PC groups. The short tube length may result in tube retraction because of dynamic movement of the AGV tube with eye movement and such complication has previously been reported [13]. And tube retraction could lead to complete retraction from the anterior chamber. However, the frequency of tube retraction did not differ between both groups in our study, which may be due to the firm fixation of the AGV plate using a non-absorbable suture. In our study, tube insertion through sclerectomy gap and short tube length left in the AC group may be a safe procedure in eyes with chronic ACG.

The hypertensive phase is defined as a transient IOP elevation associated with an encapsulated bleb in the early postoperative period, which peaked in the first month and had stabilized by 6 months after the operation. The mechanism of hypertensive phase is presumably due to inflammatory mediators related congested, thickened, encapsulated bleb around the plate of the implant and cause increased resistance to aqueous flow. It has been reported to be more prevalent in eyes after AGV implantation [14,15], which may be related to the plate surface, the biomaterial or the absence of tube ligation. Pakravan [16] reported that early aqueous suppressant treatment in AGV implantation could reduce hypertensive phase frequency, which may be due to a lower concentration of inflammatory mediators reaching the tissues surrounding the AGV plate. In our study, mean IOP elevated in the PC group at 1 month, representing the hypertensive phase. In contrast, no such elevation was observed in the AC group. We believe such difference is because of sclerectomy. In the PC group, the aqueous humor accompanied with inflammatory mediators would be drained to the area around AGV plate directly and resulted in hypertensive phase. And such condition may not happen in the AC group because the aqueous humor could be drained through sclerectomy gap to peri-limbal area instead of posterior conjunctiva around AGV plate. Moreover, the sclerectomy gap could control IOP via the filtering function at the early postoperative period. Consistent with the previous theory [16], a lower concentration of inflammatory mediators around AGV plate may led to a thinner and looser Tenon’s capsule, accounting for the noted absence of a hypertensive phase in the AC group.

The most common early postoperative complication in our study was transient hyphema (14.0%), followed by flat chamber/hypotony (4.7%). In previous studies of glaucoma drainage devices, hyphema was reported in 13–16.9% [17,18] and shallow anterior chamber/hypotony in 3–32% of cases [2,3,8,18,19]. In our study, the higher rate of hyphema in the PC group may be explained by the blind puncture that we cannot observe the needling direction when puncturing the posterior chamber. Such blind puncture could result in iris or ciliary body trauma. In flat chamber/hypotony, there was no significant difference between both groups. However, the frequency of viscoelastic anterior chamber reformation was relative higher in the AC group (0.9 ± 0.7 in AC group vs. 0.4 ± 0.8 in PC group; *p* < 0.05). The higher rate of AC group reformation may be explained by sclerectomy gap, which could prevent hypertensive phase via the filtering function at the early postoperative period. However, the frequency of viscoelastic AC reformation is low and no severe complications like suprachoroidal effusion and suprachoroidal hemorrhage were noted in both groups. Thus, we believe that sclerectomy is a safe procedure for glaucoma surgeon. Fortunately, these two early complications (hyphema and flat chamber/hypotony) could be treated by closely observation and in-office procedure. Besides, two patients of tube/plate exposure were noted at the early following period and slit-lamp examination revealed unhealthy conjunctiva with pale appearance and sloughed conjunctival sutures. The possible reasons of such early post-operative period complication may be due to inadequate conjunctival sutures and poor conjunctival healing process. Fortunately, the conjunctiva of the two patients could be re-sutured under topical anesthesia. There was no significant difference in late complications or in the preoperative to postoperative change in vision between the two groups. In addition, postoperative strabismus was not observed in our study and we believe this is because small size AGV implantation prevented direct trauma to the extraocular muscle.

There are some limitations to our study, including the small sample size, retrospective study design, heterogeneity of the study cohort, incomplete visual field data and the variability of follow-up periods. And the vision changes were established according to the patients’ subjective responses, which could not quantify the vision changes. Besides, the decision of small size AGV was made by surgeon during surgery by the conjunctival scarring condition and surgical view, other objective parameters like axial length, orbital computed tomography (CT) to prove shallow orbit were not collected from the patients. Above parameters may be considered in future prospective studies to provide more precise outcome. One important limitation is that all enrolled subjects in the present study were Asian, so the present results may not be applicable for other race.

## 5. Conclusions

In conclusion, in the 43 adult chronic adult ACG patients included in this study, small size AGV insertion showed an overall cumulative probability of success at 24 months were 67.4% for qualified success and 39.5% for complete success, indicating that small size AGV insertion could be effective at lowering IOP. Besides, tube insertion into anterior chamber with sclerectomy may prevent the hypertensive phase in the early postoperative period.

## Figures and Tables

**Table 1:** Demographic Characteristics of the Patient Cohort.

	AC Group (*n* = 24)	PC Group (*n* = 19)	*p*
Age (Years)	54.7 (95% CI: 48.7–60.6)	58.8 (95% CI: 51.8–65.8)	0.2397
Sex			
Male	8	10	0.2304
Female	16	9	
Eye			
Right	12	6	0.3510
Left	12	13	
Lens status			
Phakic	4	0	0.1175
Pseudophakic	20	19	
Mean pre-operative intraocular pressure (mmHg)	36.8 (95% CI: 32.4–41.1)	42.2 (95% CI: 37.2–47.2)	0.1036
Mean pre-operative glaucoma medications	2.7 (95% CI: 2.4–3.0)	3.1 (95% CI: 2.8–3.5)	0.0811
Previous surgery			
Trabeculectomy	24	19	
Cataract surgery	20	19	0.1175
Vitrectomy	0	0	
Disease type			
PACG	10	4	0.5623
UG	3	4	
NVG	6	4	
PKG	4	6	
Other	1	1	
Follow-up (Months)	29.5 (95% CI: 24.8–34.2)	27.2 (95% CI: 23.5–30.8)	0.4350

PACG = primary angle closure glaucoma; UG = uveitic glaucoma; NVG = neovascular glaucoma; PKG = post keratoplasty glaucoma; AC: anterior chamber; PC: posterior chamber; CI: confidence interval.

**Table 2:** Mean Intraocular Pressures (IOP) of the Patient Cohort (mmHg).

	Total (*n* = 43)	AC Group (*n* = 24)	PC Group (*n* = 19)	*p*
Preoperative (Day 0)	39.2 (95% CI: 35.9–42.4)	36.8 (95% CI: 32.4–41.1)	42.2 (95% CI: 37.2–47.2)	0.1036
First day (Day 1)	9.4 (95% CI: 8.6–10.2)	8.5 (95% CI: 7.6–9.4)	10.6 (95% CI: 9.3–11.9)	0.0160 *
Seventh day (Day 7)	12.1 (95% CI: 10.9–13.3)	12.5 (95% CI: 10.9–14.2)	11.6 (95% CI: 9.72–13.4)	0.5067
First month (Day 30)	15.6 (95% CI: 13.8–17.4)	12.1 (95% CI: 11.2–13.1)	20.0 (95% CI: 17.0–23.0)	<0.0001 *
Third month (Day 90)	16.1 (95% CI: 14.3–17.8)	15.6 (95% CI: 12.8–17.5)	17.2 (95% CI: 14.4–20.0)	0.1135
Sixth month (Day 180)	15.7 (95% CI: 14.9–16.6)	15.5 (95% CI: 14.4–16.6)	16.1 (95% CI: 14.7–17.4)	0.7197
First year (Day 360)	19.0 (95% CI: 17.6–20.3)	19.6 (95% CI: 17.5–21.7)	18.1 (95% CI: 16.5–19.7)	0.5049
Second year (Day 720)	20.2 (95% CI: 17.8–22.5)	19.5 (95% CI: 16.5–22.5)	21.1 (95% CI: 17.1–25.2)	0.5236

Addressed IOP data includes successful and failed patients. AC: anterior chamber; PC: posterior chamber; CI: confidence interval. *: *p* < 0.05.

**Table 3:** Final Vision Changes of the Patient Cohort

	Total (*n* = 43)	AC Group (*n* = 24)	PC Group (*n* = 19)	*p*
Worse	13	8	5	0.8601
Better	7	4	3	
No change	23	12	11	

AC: anterior chamber; PC: posterior chamber.

**Table 4:** Mean Number of Antiglaucoma Medications of the Patient Cohort.

	Preoperative	Postoperative	*p*
Total (*n* = 43)	2.9 (95% CI: 2.7–3.1)	1.5 (95% CI: 1.0–1.9)	<0.0001 *
AC group (*n* = 24)	2.7 (95% CI: 2.4–3.0)	1.2 (95% CI: 0.6–1.8)	<0.0001 *
PC group (*n* = 19)	3.1 (95% CI: 2.8–3.5)	1.8 (95% CI: 1.2–2.5)	0.0024 *

AC: anterior chamber; PC: posterior chamber; CI: confidence interval. *: *p* <0.05.

**Table 5:** Post-operative Complications in the Patient Cohort.

	Total (*n*, %)	AC Group (*n*, %)	PC Group (*n*, %)	*p*
Hyphema	6, 14.0%	1, 4.2%	5, 26.3%	0.0374 *
Flat chamber/Hypotony	2, 4.7%	1, 4.2%	1, 5.3%	0.8654
Tube or plate exposure	2, 4.7%	1, 4.2%	1, 5.3%	0.8654
Tube retraction	3, 7.0%	1, 4.2%	2, 10.5%	0.4163
Encapsulated bleb	6, 14.0%	3, 12.5%	3, 15.8%	0.7572
Endophthalmitis	1, 2.3%	1, 4.2%	0, 0%	0.3680
Bullous keratopathy	3, 7.0%	2, 8.3%	1, 5.3%	0.6947
Strabismus	0, 0%	0, 0%	0, 0%	

AC: anterior chamber; PC: posterior chamber. *: *p* <0.05.

## Data Availability

The data presented in this study are available on reasonable request from the corresponding author.
